# Characterization of community-acquired *Clostridioides difficile* strains in Israel, 2020–2022

**DOI:** 10.3389/fmicb.2023.1323257

**Published:** 2023-12-19

**Authors:** Orna Schwartz, Hanan Rohana, Maya Azrad, Anna Shor, Nir Rainy, Yasmin Maor, Lior Nesher, Orli Sagi, Shifra Ken-Dror, Peter Kechker, Avi Peretz

**Affiliations:** ^1^Azrieli Faculty of Medicine, Bar Ilan University, Safed, Israel; ^2^Clinical Microbiology Laboratory, The Edith Wolfson Medical Center, Holon, Israel; ^3^Sackler Faculty of Medicine, Tel Aviv University, Tel Aviv, Israel; ^4^Clinical Microbiology Laboratory, Tzafon Medical Center, Poriya, Israel; ^5^Shamir Medical Center, Be’er Ya’akov, Israel; ^6^Infectious Diseases Unit, The Edith Wolfson Medical Center, Holon, Israel; ^7^Infectious Diseases Institute, Soroka University Medical Center, Be’er Sheba, Israel; ^8^Faculty of Health Sciences Ben-Gurion University of the Negev, Be’er Sheba, Israel; ^9^Microbiology Laboratory, Soroka University Medical Center, Be’er Sheba, Israel; ^10^W. Hirsch Regional Microbiology Laboratory, Clalit Health Services, Haifa, Israel

**Keywords:** *C* *. difficile*, CDI, community-acquired *C. difficile* infection, MLST, clade

## Abstract

**Background:**

The prevalence of community-acquired *Clostridioides difficile* infection (CA-CDI) has been rising, due to changes in antibiotics prescribing practices, emergence of hypervirulent strains and improved diagnostics. This study explored CA-CDI epidemiology by examining strain diversity and virulence factors of CA-CDI isolates collected across several geographical regions in Israel.

**Methods:**

Stool samples of 126 CA-CDI patients were subjected to PCR and an immunoassay to identify toxin genes and proteins, respectively. Toxin loci PaLoc and PaCdt were detected by whole-genome sequencing (WGS). Biofilm production was assessed by crystal violet-based assay. Minimum inhibitory concentration was determined using the Etest technique or agar dilution. WGS and multi-locus sequence typing (MLST) were used to classify strains and investigate genetic diversity.

**Results:**

Sequence types (ST) 2 (17, 13.5%), ST42 (13, 10.3%), ST104 (10, 8%) and ST11 (9, 7.1%) were the most common. All (117, 92.8%) but ST11 belonged to Clade 1. No associations were found between ST and gender, geographic area or antibiotic susceptibility. Although all strains harbored toxins genes, 34 (27%) produced toxin A only, and 54 (42.9%) strains produced toxin B only; 38 (30.2%) produced both toxins. Most isolates were biofilm-producers (118, 93.6%), primarily weak producers (83/118, 70.3%). ST was significantly associated with both biofilm and toxin production.

**Conclusion:**

*C. difficile* isolates in Israel community exhibit high ST diversity, with no dominant strain. Other factors may influence the clinical outcomes of CDI such as toxin production, antibiotic resistance and biofilm production. Further studies are needed to better understand the dynamics and influence of these factors on CA-CDI.

## 1 Introduction

For years, *Clostridioides difficile* (*C. difficile*) infection (CDI) has been considered as a leading cause of healthcare-associated (HA) infections. In recent years, there is growing evidence for CDI among individuals with no prior history of hospitalizations or exposure to healthcare settings, indicating an upward trend in community-acquired CDI (CA-CDI) (Fu et al., 2021). CDI is a toxin-mediated disease, mainly caused by the production of toxin A (TcdA) and toxin B (TcdB), encoded by the genes *tcd*A and *tcd*B, respectively, which are part of a pathogenicity locus (PaLoc) (Kuehne et al., 2010). These toxins catalyze the inactivation of small GTPases (such as Rho, Rac and Ras), which results in cytoskeletal disorganization, hemorrhage and the release of fluid into the intestinal tract, causing the watery diarrhea that is characteristic of CDI (Poutanen and Simor, 2004; Voth and Ballard, 2005). Toxin B is responsible for inhibiting RNA synthesis, phagocytosis, immune cell migration and cytokine production. In addition to these significant toxins, the bacterium may harbor some other virulence factors, including biofilm formation capacity and CDT binary toxin production, a toxin that is encoded by the genes *cdt*A and *cdt*B, which are part of the CdtLoc locus (Dapa et al., 2013; Gerding and Lessa, 2015).

The most significant risk factor for CDI is the use of antibiotics, which disrupts the indigenous gastrointestinal microbiota (Rohana et al., 2020). The main antimicrobial agents contributing to CDI are fluoroquinolones, clindamycin and cephalosporins (Asempa and Nicolau, 2017). The main risk factors for CA-CDI include water and food contaminations, antimicrobial and acid-suppression medications overuse, epidemic strains, and asymptomatic carriage (Gupta and Khanna, 2014). Some *C. difficile* strains are predominant in community settings, suggesting the potential for animal involvement or transmission (Ofori et al., 2018).

Patients diagnosed with CA-CDI are usually younger compared to HA-CDI patients, with some studies reporting on a difference of up to 20 years (Khanna et al., 2012; Lessa et al., 2015). For example, Khanna et al. (2012) reported on a median age of 50 versus 72 years, respectively. Additionally, lower mortality rates were reported among patients with CA-CDI (Banks et al., 2018).

Although strain identification is crucial for epidemiological surveillance, it is not part of routine practice. Instead, CDI diagnosis typically relies on algorithms, which include molecular biology techniques to detect the presence of toxin genes (Florea et al., 2014). This approach is favored in healthcare facilities due to its short turn-around time, as culturing *C. difficile* can take up to 48 h. Apart from its application for characterization of *C. difficile* isolates, molecular typing is also used to investigate the changing epidemiology of CDI. Multi-locus sequence typing (MLST) is a method based on nucleotide sequences of several housekeeping genes. The consensus scheme for *C. difficile* is based on seven genes, with a sequence type (ST) number assigned to each combination of alleles (Muñoz et al., 2017). Another approach is ribotyping in which strain-specific differences are identified based on variability on the number and length of interspace regions between ribosomal RNA 16S and 23S genes (Krutova et al., 2018).

Molecular typing studies demonstrated that CA-CDI strains have a diverse epidemiology, with some features common with but also different from those of HA-CDI strains (Shin et al., 2011; Dumyati et al., 2012; Fu et al., 2021). For example, ribotype 106 was detected among both CA-CDI and HA-CDI patients, while ribotype 002 was found in CA-CDI patients (Fu et al., 2021). This study aimed to investigate the distribution and the strain types of isolates originating from CA-CDI patients in several geographic areas in Israel during 2020–2022. Additionally, it aimed to characterize several *C. difficile* virulence factors, including toxins, biofilm production capacity, and antibiotic susceptibility. The data provide a comprehensive image of the epidemiology of CA-CDI in Israel.

## 2 Materials and methods

### 2.1 Study population and sample collection

The study included stool samples from patients who acquired CDI in the community and were hospitalized in one of four medical centers in Israel between 2020 and 2022. CA-CDI was determined as CDI that was developed within the first 48 h of hospitalization. The medical centers represent different geographic areas of Israel: Tzafon Medical Center, Poriya and W. Hirsch Regional Microbiology Laboratory Clalit Health Services, Haifa, are located at north Israel, the Edith Wolfson Medical Center, Holon, is located in the center of Israel, and Soroka University Medical Center, Be’er Sheva, is located in southern Israel.

Clinical and demographic data including age, gender and death within 30 days of hospitalization, were collected from patient medical records.

All CDI cases were confirmed by stool examination for toxin B, binary toxin, and tcdC deletion using the GeneXpert *C. difficile* BT PCR assay (Cepheid, Sunnyvale, CA, United States). The study was approved by local Ethics (Helsinki) Committee of each medical center (POR-0085-15, WOMC-0115-20, SOR-0307-20). These committees granted a waiver for informed consent.

### 2.2 Bacterial isolation and identification

Stool samples were inoculated on chromID^™^ *C. difficile* (CDIF) (BioMérieux, Durham, NC), a selective growth agar medium, and incubated at 37°C under anaerobic conditions (10% CO_2_, 10% H_2_; and 80% N_2_), in a Bactron EZ 300 anaerobic chamber (Sheldon manufacturing, Cornelius, United States) for 48 h. *C. difficile* colonies were identified by their asymmetrical shape and black color. For conclusive identification, matrix-assisted laser desorption ionization-time of flight (MALDI-TOF) mass spectrometry was employed using the Bruker Biotyper system (Bruker Daltonics, Bremen, Germany) (Singhal et al., 2015). All isolates were stored as beads at −80°C for further analysis.

### 2.3 Whole-genome sequencing and multi-locus sequence typing of bacterial isolates

#### 2.3.1 DNA extraction

Total genomic DNA was extracted from bacterial isolates using the MagCore^®^ Genomic DNA. Bacterial Kit (ATRIDAB.V., Amersfoort, Nederlands) and the MagCore^®^ automated extraction instrument (RBCBioscience, New Taipei, Taiwan), according to manufacturer instructions.

#### 2.3.2 Library preparation and whole-genome sequencing

Following DNA cleanup, whole-genome sequencing was carried out using the Illumina DNA Prep kit (Illumina, Inc., San Diego, CA, United States) according to the manufacturer’s protocol. Specifically, samples were processed for tagmentation and adapter ligation using IDT for Illumina Nextera UD Indexes Set A, B, C, D. Further, enrichment and cleanup were performed according to the manufacturer’s instructions.

Libraries were pooled into 8 pools of 40 samples each. DNA of pooled samples was quantified by the Qubit 4.0 fluorometer using HS DS DNA kit (Thermo Fisher Scientific) and fragment sizes were determined by the TapeStation 4150 via DNA HS D1000 kit (Agilent Technologies, Santa Clara, CA, United States). The pooled libraries were then brought to a concentration of 4 nM and 5 μL of each pool was then combined in a fresh microcentrifuge tube. For sequencing, pooled libraries were denatured and neutralized with 0.2 N NaOH and 400 mM Tris-HCL (pH 8). Dual indexed paired-end sequencing with 149 bp read length was carried out on the NovaSeq6000 platform (Illumina, Inc., San Diego, CA, United States).

Resulting FastQ files were subjected to analysis (alignment and QA/QC) in Partek^®^ Flow^®^, using the reference sequence of *C. difficile* strain 630 (NC_009089). A second analysis was performed in the Genius program, using BAM files (created in Partek Flow) of the failed samples, to visually observe the depth and quality of the missing genes.

#### 2.3.3 MLST analysis

To determine the sequencing type, the PubMLST *C. difficile* database^1^ was used to analyze the sequences of 7 housekeeping genes (adk, atpA, dxr, glyA, recA, sodA, tpi) in each isolate, as previously described (Griffiths et al., 2010). The gene sequences were also used to assess the evolutionary relationship of different strains, enabling the identification of clades, i.e., phylogenetic lineages.

MUSCLE-aligned concatenated allele sequences (concatenated and aligned in Geneious Prime) were used as input for IQ-TREE v2.2.2.6 (doi: 10.1093/molbev/msaa015), along with ModelFinder (doi: 10.1038/nmeth.4285) to generate the maximum likelihood phylogenetic tree. The tree was visualized with an online tool for phylogenetic tree display and annotation iTOL (doi: 10.1093/nar/gkab301).

#### 2.3.4 Toxin gene analysis

Whole-genome sequencing data were used to detect the toxin loci PaLoc and PaCdt. These loci were examined by comparing the average coverage of the genes ***tcd***A, ***tcd***B, ***cdt***A and ***cdt***B among the alignments of each sample to *C. difficile* strain 630 (which is A + B + CDT−) and *C. difficile* strain 196 (which is A + B + CDT+).

### 2.4 Toxin protein detection

*Clostridioides difficile* toxins were detected in fecal samples using the CerTest *C. difficile* GDH + Toxin A + B combo card test kit (Certest Biotec, S.L, Zaragoza, Spain), in accordance with the manufacturer’s protocol. This chromatographic immunoassay allows for the simultaneous qualitative detection of *C. difficile* antigens toxin A, toxin B, and glutamate dehydrogenase (GDH), an enzyme abundantly produced by all *C. difficile* strains (Gateau et al., 2018).

### 2.5 Microtiter plate assay for the assessment of biofilm production

Biofilm production was assessed using a microtiter plate, as previously described (Hammond et al., 2014). Each *C. difficile* strain was cultured in brain heart infusion agar supplemented with yeast extract +0.1% L-cysteine (BHIS), and incubated at 37°C for 24 h under anaerobic conditions. A sample of the suspension was diluted to an optical density (OD_600nm_) of 0.8 and then diluted 1:100 in BHIS broth. A 200 μL portion of each diluted inoculum was dispensed into wells of a 96-well plate. The biofilm-forming ATCC^®^ BAA-1382 *C. difficile* strain (strain 630) served as a positive control and BHIS as a negative control. The plate was incubated at 37°C for 24 h, in an anaerobic cabinet. Subsequently, the spent medium was aspirated, the wells were washed twice with phosphate-buffered saline (PBS) (Oxoid, Cambridge, United Kingdom), and then 200 μL of a 0.25% (w/v) aqueous crystal violet solution were added to each well. After a 5 min incubation, the wells were washed with PBS eight times and allowed to air-dry. To dissolve the dye from the adherent cells, 200 μL of an ethanol:acetone (1:1) solution was added to each well. Absorbance was measured within 5 min at a wavelength of 570 nm using an ELISA reader (Multiskan Go, Fisher-Scientific Ltd., Vantaa, Finland). The cut-off OD_570nm_ (ODc) was determined as three standard deviations above the mean OD of the negative control. The isolates were classified as previously described (Stepanovic et al., 2000): non-producers—isolates with OD < ODc, weak producers—isolates with ODc < OD ≤ 2× ODc, moderate producers—2× ODc < OD ≤ 4× ODc and strong producers - OD > 4× ODc. The experiment was repeated 3 times, with triplicates of each strain tested in each experiment.

### 2.6 Antimicrobial susceptibility testing

The minimum inhibitory concentration (MIC) was determined for several antibiotics using the Etest technique. Following isolation, *C. difficile* colonies were cultured in thioglycollate broth medium (Becton Dickinson, Heidelberg, Germany) until reaching a turbidity of 1.0 McFarland. The suspension was spread on Brucella blood agar (Hy Laboratories, Rehovot, Israel) and a gradient Etest strip (bioMérieux, Durham, NC) containing vancomycin, metronidazole or moxifloxacin was placed on each plate.

The plates were then incubated under anaerobic conditions at 37°C for 48 h. MIC was determined for each antibiotic and strains were classified as susceptible or resistant based on the guidelines of the European Committee on Antimicrobial Susceptibility Testing (EUCAST). According to these standards, resistance of *C. difficile* to vancomycin and metronidazole is defined with MIC >2 μg/mL, while reduced susceptibility to moxifloxacin is determined with MIC >4 μg/mL as an epidemiological cut-off, (European Committee on Antimicrobial, 2017).

### 2.7 Susceptibility of *Clostridioides difficile* isolates to fidaxomicin

Agar dilution was performed to assess susceptibility of *C. difficile* isolates to fidaxomicin, according to the procedures of the Clinical and Laboratory Standards Institute (CLSI-M11-9th) (Meng et al., 2021). Fidaxomicin (Sigma-Aldrich, Missouri, United States) was dissolved in dimethyl sulfoxide (DMSO) and then further diluted with distilled water (Thorpe et al., 2019). The diluted reagent was then added to Brucella Agar +5% Defibrinated Sheep Blood (Hy Laboratories), to final concentrations of 0.03–32 μg/mL. *C. difficile* colonies were suspended in thioglycollate broth medium (Becton Dickinson) to 0.5 McFarland turbidity. The suspension was then added as a spot to plates containing different concentration of fidaxomicin, that were incubated at 35°C for 48 h, under anaerobic conditions. Following incubation, plates were screened for presence of growth. The MIC was defined as the lowest concentration of the agent that inhibited growth of the bacteria by 90%.

### 2.8 Statistical analysis

The Fisher exact test and Pearson’s chi-squared test were applied to analyze differences between probabilities of categorical variables [including differences in characteristics of patients (except age) or bacteria between STs, and differences between antibiotic resistance rates between the years or between STs. The independent samples Kruskal–Wallis rank sum test was performed to analyse differences in mean patients age or mean bacterial MIC between different STs. A *p*-value <0.05 was considered statistically significant. The data were analyzed using the RStudio^®^ version 2021.09.0 Build 351.

## 3 Results

### 3.1 Demographic characteristics of patients

In total, 126 CDI patients were enrolled in this study, 63.5% (80/126) of whom were female. The average age of the study population was 64.9 ± 22.7 years. Most samples were collected from patients residing in the northern region of the country (59.5%; 75/126), while 20.6% (26/124) and 19.8% (25/126) were from the central and southern regions, respectively. The 30 days mortality rate was 8.0% (Table 1).

**Table 1:** Demographic characteristics of CA-CDI patients.

Patient characteristic	
**Age (years)**	
Mean	64.90
SD	22.65
**Gender (*n*, %)**	
Female	80 (63.5)
Male	46 (36.5)
**Location (*n*, %)**	
North	75 (59.5)
Center	26 (20.6)
South	25 (19.8)
**30 days mortality (*n*, %)**	
Survived	116 (92.1)
Died	10 (7.9)

### 3.2 Distribution of STs

**Figure 1:**
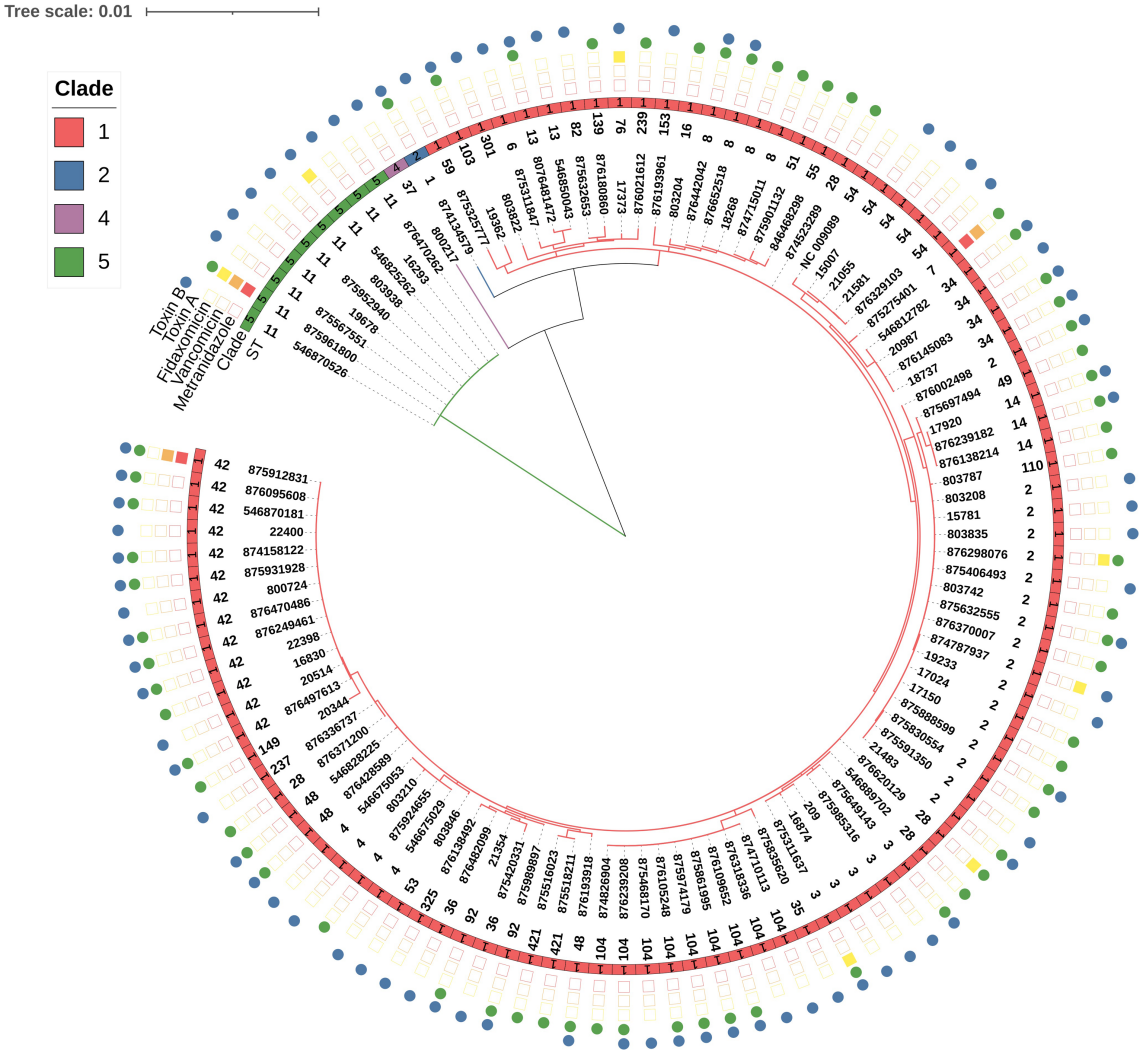
Phylogenetic tree of *C. difficile* strains, based on whole-genome sequencing. The STs are indicated after the strain labels. MLST clades are marked with colored squares: Clade 1 (red), Clade 2 (blue), Clade 4 (purple) and Clade 5 (green). NC_009089 was used as a reference genome for alignment. Tree scale: 0.001. The presence of toxin A and B proteins is marked by green and blue circles, respectively. Resistance to metronidazole or vancomycin is marked by a red or orange square, respectively. A yellow square indicates fidaxomicin MIC≥4 μg/mL.

Due to inconclusive results for 11 isolates, MLST analysis was performed on 91.3% (115/126) of the samples (Figure 1). Isolates were categorized into four major ST groups, each of which included at least six isolates, and an additional group called “others,” which included 35 different STs (with <6 isolates per ST). The four most frequent STs were ST2 (13.5%, 17/126), ST42 (10.3%, 13/126), ST104 (8%, 10/126) and ST11 (7.1%, 9/126), with the first three belonging to Clade 1 and ST11 belonging to Clade 5. All but two isolates in the “Others” group, belonged to Clade 1; the outliers belonged to ST1/ Clade 2 and to ST37/Clade 4 (Figure 1).

### 3.3 Demographic characteristics of CDI patients and ST

No significant associations between ST and demographic characteristics of patients (Table 2).

**Table 2:** Associations between patient demographics and *C. difficile* ST.

	ST104 (*N* = 10)	ST11 (*N* = 9)	ST2 (*N* = 17)	ST42 (*N* = 13)	Inconclusive (*N* = 11)	Other (*N* = 66)	Total (*N* = 126)	*p*-value
Age (years)								0.218
Mean	72.80	73.67	55.12	69.46	69.55	63.36	64.90	
SD	18.73	23.08	25.56	18.78	19.08	23.12	22.65	
Gender (*n*, %)								0.072
Female	6 (60)	8 (88.9)	9 (52.9)	5 (38.5)	5 (45.5)	47 (71.2)	80 (63.5)	
Male	4 (40)	1 (11.1)	8 (47.1)	8 (61.5)	6 (54.5)	19 (28.8)	46 (36.5)	
Location (*n*, %)								0.537
North	9 (90)	5 (55.6)	8 (47.1)	8 (61.5)	5 (45.5)	40 (60.6)	75 (59.5)	
Center	0 (0)	2 (22.2)	5 (29.4)	4 (30.8)	2 (18.2)	13 (19.7)	26 (20.6)	
South	1 (10)	2 (22.2)	4 (23.5)	1 (7.7)	4 (36.4)	13 (19.7)	25 (19.8)	
30 days mortality								0.705
Survival	9 (90)	9 (100)	17 (100)	12 (92.3)	10 (90.9)	59 (89.4)	116 (92.1)	
Death	1 (10)	0 (0)	0 (0)	1 (7.7)	1 (9.1)	7 (10.6)	10 (7.9)	

### 3.4 Bacterial virulence factors

Although all isolates harbored the genes for both toxins, not all isolates produced both toxins. Toxin A protein was detected in 27.0% (34/126) of the strains, Toxin B protein was detected in 42.9% (54/126) of the strains, and 30.2% (38/126) of the isolates were positive for both toxins (Table 3). Overall, 93.7% (118/126) of isolates produced biofilm. Most of the biofilm-producing isolates were weak producers (65.9%, 83/118), while 19.0% (24/118) were moderate producers and 8.7% (11/118) were strong producers.

**Table 3:** Bacterial virulence factors.

Strain characteristic	*N* (%)
**Toxin protein detection**	
Toxin A	34 (27.0)
Toxin B	54 (42.9)
Toxins A + B	38 (30.2)
**Biofilm O.D.**	
Mean	0.18
SD	0.14
**Biofilm-producing capacity**	
Non-producer	8 (6.3)
Weak	83 (65.9)
Moderate	24 (19.0)
Strong	11 (8.7)

### 3.5 Associations between bacterial virulence factors and ST

**Figure 2:**
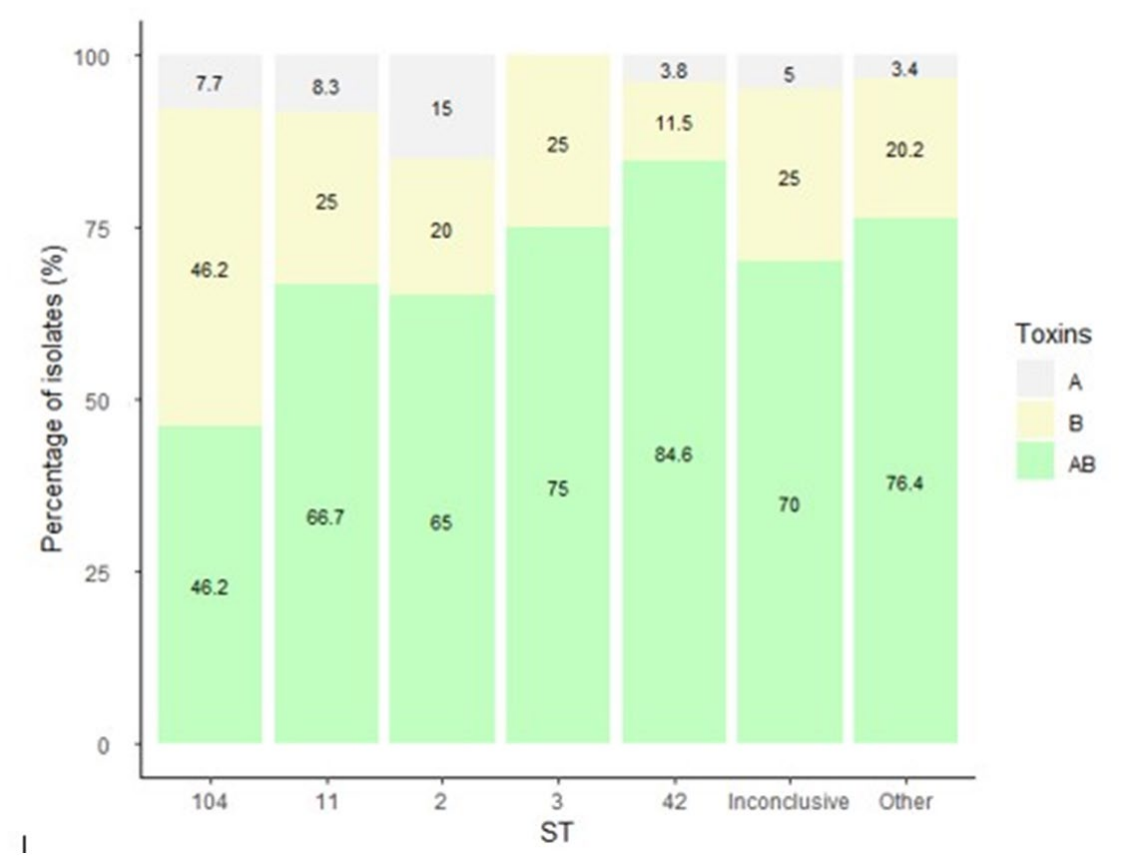
Distribution of *C. difficile* toxin expression by isolate ST. The figure presents the percentage of isolates that produced either toxin A, B or both toxins, by ST. *C. difficile* toxins were detected in fecal samples using the CerTest *Clostridium difficile* GDH + Toxin A + B combo card test kit.

No significant association was found between ST and 30 days mortality. Nevertheless, a significant association was found between ST and toxin production (*p* = 0.01). Among the ST11 isolates, 11.1% (1/9) produced toxin A and 88.9% (8/9) produced toxin B. In contrast, 7.7% (1/13) of ST42 isolates produced toxin A, 23.1% (3/13) and toxin B, while 69.2% (9/13) produced both toxins (Table 4 and Figure 2). Concerning biofilm production, all ST11 isolates (100%, 9/9) and 92.3% (12/13) of ST42 isolates were weak biofilm producers. Among the non-producers, ST104 was the most predominant (87.5%, 7/8; Table 2).

**Table 4:** Associations between bacterial characteristics and ST.

	ST104 (*N* = 10)	ST11 (*N* = 9)	ST2 (*N* = 17)	ST42 (*N* = 13)	Inconclusive (*N* = 11)	Other (*N* = 66)	Total (*N* = 126)	*p*-value
Toxin expressed (*n*, %)								**0.010**
A	1 (10)	1 (11.1)	6 (35.3)	1 (7.7)	5 (45.5)	20 (30.3)	34 (27)	
B	4 (40)	8 (88.9)	7 (41.2)	3 (23.1)	4 (36.4)	28 (42.4)	54 (42.9)	
A + B	5 (50)	0 (0)	4 (23.5)	9 (69.2)	2 (18.2)	18 (27.3)	38 (30.2)	
Biofilm-producing capacity (*n*, %)								**<0.001**
Non-producer	7 (70)	0 (0)	0 (0)	0 (0)	0 (0)	1 (1.5)	8 (6.3)	
Weak	2 (20)	9 (100)	4 (23)	12 (92.3)	7 (63.6)	49 (74.2)	83 (65.9)	
Moderate	0 (0)	0 (0)	12 (70.6)	1 (7.7)	3 (27.3)	8 (12.1)	24 (19)	
Strong	1 (10)	0 (0)	1 (5.9)	0 (0)	1 (9.1)	8 (12.1)	11 (8.7)	

Bold numbers represent statistically significant associations.

### 3.6 Antibiotics susceptibility patterns

**Figure 3:**
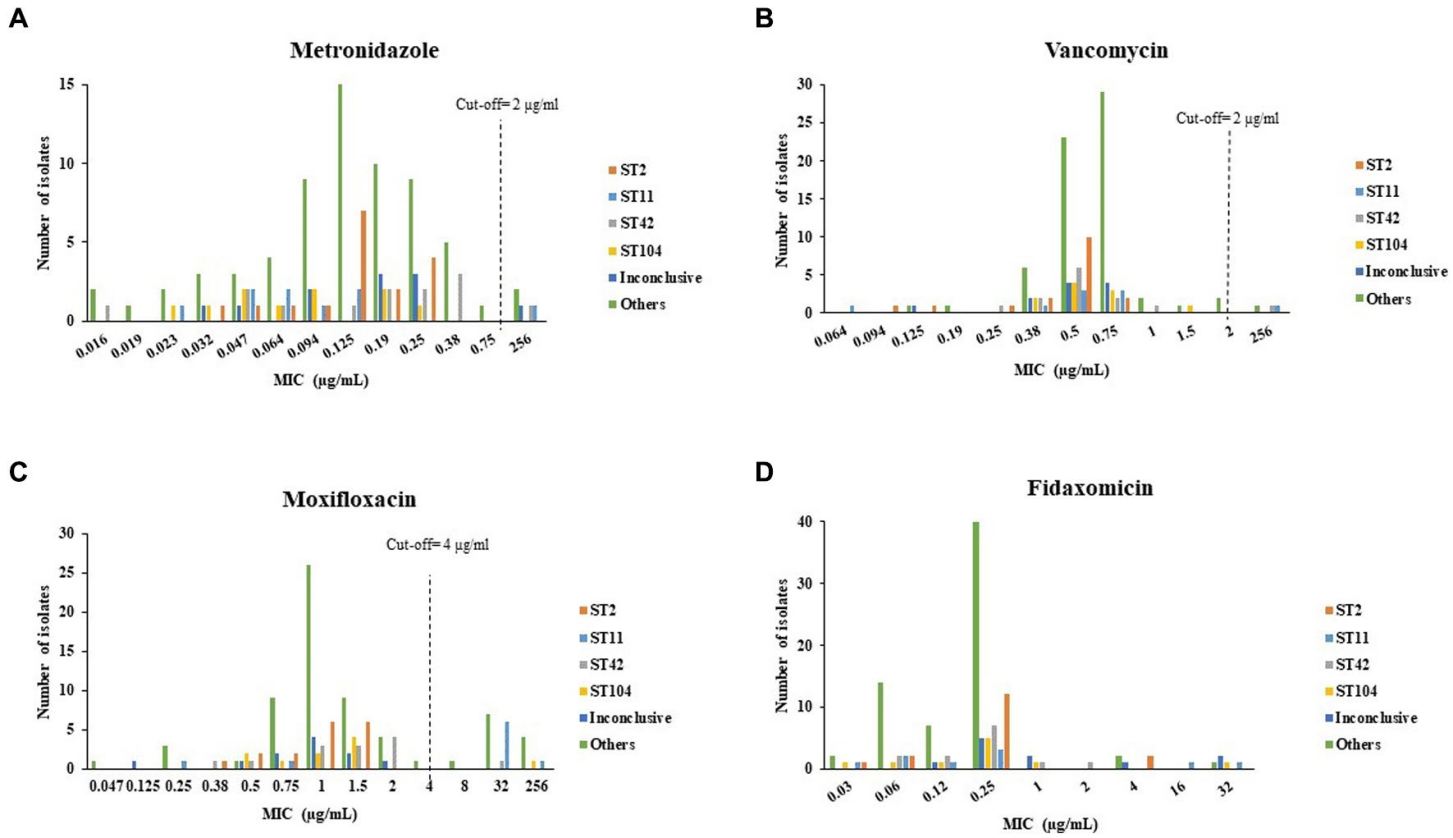
Distribution of **(A)** metronidazole, **(B)** vancomycin, **(C)** moxifloxacin, **(D)** fidaxomicin minimum inhibitory concentration (MIC) by *C. difficile* isolate ST. Antibiotic susceptibility was tested by the Etest method for metronidazole, vancomycin and moxifloxacin, and by the agar dilution assay for fidaxomicin.

Figure 3 present the distribution of antibiotics MIC by isolate ST. The highest MIC values for metronidazole were measured for isolates that belonged to ST11, ST42, the inconclusive group and the Others group (Figure 3A). For vancomycin, isolates of ST11, ST42 and the Others group had the highest MIC (Figure 3B). For moxifloxacin, isolates of ST104, the inconclusive group and the Others group had the highest MIC (Figure 3C). Isolates of ST11, ST42, the Others group and of the inconclusive group had the highest MIC for fidaxomicin (Figure 3D).

**Figure 4:**
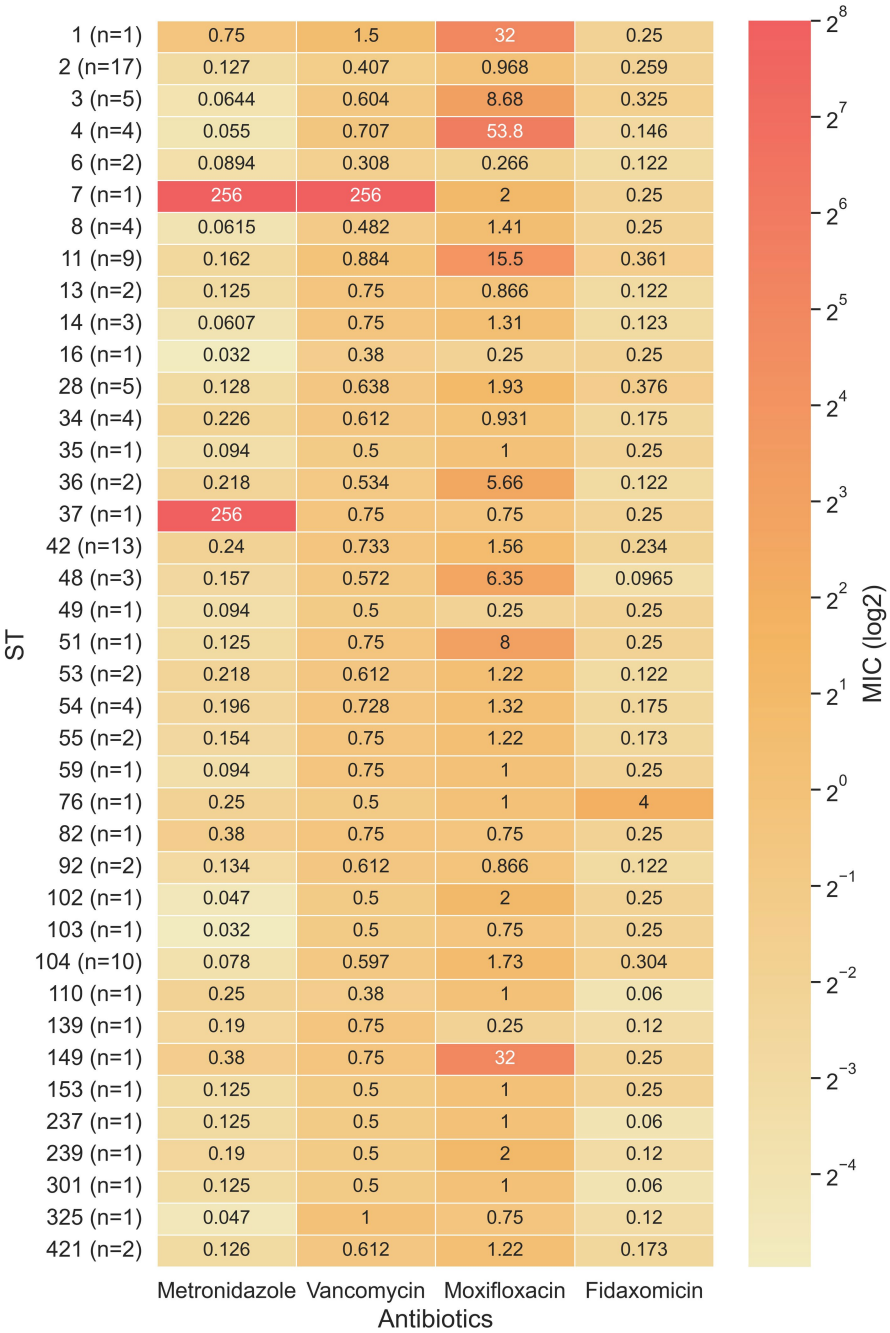
Heat map of the minimum inhibitory concentration (MIC) for four antibiotics in relation to 39 sequence types. When a sequence type covered multiple samples, the geometric mean was used to calculate the average MIC.

ST103 and ST16 had the lowest metronidazole geometric mean MIC (GM-MIC) (0.032 μg/mL for both), while ST37 and ST7 had the highest metronidazole GM-MIC (256 μg/mL for both). For vancomycin, the lowest GM-MIC (0.308 μg/mL) was measured for ST6, while ST7 had the highest GM-MIC (256 μg/mL). For moxifloxacin, ST16, ST49 and S139 shared the lowest GM-MIC (0.25 μg/mL) while ST4 had the highest GM-MIC (53.9 μg/mL). Fidaxomicin GM-MIC was lowest for ST110, ST237 and ST301 (0.06 μg/mL), while ST76 had the highest GM-MIC (4 μg/mL) (Figure 4).

No significant differences in susceptibility to metronidazole and vancomycin were noted between samples collected over the 3 years of the study (Table 5).

**Table 5:** Antibiotic susceptibility rates by year.

Antibiotic	2020 (*N* = 30)	2021 (*N* = 62)	2022 (*N* = 34)	Total (*N* = 126)	*p*-value
Metronidazole (*n*, %)					0.996
Susceptible	29 (96.7)	60 (96.8)	33 (97.1)	122 (96.8)	
Resistant	1 (3.3)	2 (3.2)	1 (2.9)	4 (3.2)	
Vancomycin (*n*, %)					0.616
Susceptible	30 (100.0)	60 (96.8)	33 (97.1)	123 (97.6)	
Resistant	0 (0.0)	2 (3.2)	1 (2.9)	3 (2.4)	

No statistically significant association was found between ST and susceptibility to metronidazole; most isolates were susceptible. A similar pattern was observed for vancomycin (Table 6).

**Table 6:** Associations between antibiotics susceptibility and isolate ST.

	ST104 (*N* = 10)	ST11 (*N* = 9)	ST2 (*N* = 17)	ST42 (*N* = 13)	Inconclusive (*N* = 11)	Other (*N* = 66)	Total (*N* = 126)	*p*-value
Metronidazole								0.365
Susceptible	10 (100.0)	8 (88.9)	17 (100.0)	12 (92.3)	10 (90.9)	65 (98.5)	122 (96.8)	
Resistant	0 (0.0)	1 (11.1)	0 (0.0)	1 (7.7)	1 (9.1)	1 (1.5)	4 (3.2)	
Vancomycin								0.340
Susceptible	10 (100.0)	8 (88.9)	17 (100.0)	12 (92.3)	11 (100.0)	65 (98.5)	123 (97.6)	
Resistant	0 (0.0)	1 (11.1)	0 (0.0)	1 (7.7)	0 (0.0)	1 (1.5)	3 (2.4)	

## 4 Discussion

Traditionally associated with healthcare facilities, CDI has now become a formidable threat in the community setting as well. This shift in epidemiology has raised concerns among healthcare professionals and researchers, as it presents new challenges in understanding the transmission and management of CDI. The current study investigated characteristics of *C. difficile* isolates obtained from patients diagnosed with CA-CDI between 2020 and 2022 across different geographic regions in Israel. Interestingly, the mean age of the study population was higher than expected (64.9 years), as patients with CA-CDI are usually younger than HA-CDI patients; according to Fu et al., the median age of HA-CDI patients is 72 years, while CA-CDI patients have a median age of 50–51.1 However, a previous comparison of the characteristics of patients in Belgium with CA- vs. HA-CDI, reported on a median age of 75 years for the CA-CDI population (Viseur et al., 2011). Most (63.5%) of the patients in the current population were women, which aligned with a previous study in which 62% of CA-CDI patients were women (Viseur et al., 2011). Further studies are needed to determine the reasons for the higher incidence of CA-CDI among women as compared to men.

The 30 days mortality rate in the current study was 7.9%. This rate is quite similar to the rate (6.9%) found in a study conducted in Quebec, Canada (Loo et al., 2005). In another study, the rate was 9.2% (Lee et al., 2018).

### 4.1 STs and virulence factors of distinct STs in the community

The main STs observed in the current community-based study were ST104, ST11, ST2 and ST42. In a study conducted on CA-CDI samples in the United States, the most frequently detected ST was ST1/NAP1 (21.7%), followed by ST20 (11.5%) and ST42 (10.9%) (Chitnis et al., 2013). These three STs were also the most common in another study conducted in United States (Lessa et al., 2015). In Europe, the most prevalent STs in the community were ST20, ST42 and ST10 (Hensgens et al., 2009; Bauer et al., 2011). Thus, there seem to be some differences in ST distribution across geographic regions. These differences may result from local transmission of strains, and maybe point that treatment regimens are inefficient in eradication of specific *C. difficile* strains.

Biofilm production was observed in 93.7% of the isolates included in the present study. A study conducted in Mexico reported a similar proportion of biofilm producers (98.1%, 100/102) (Tijerina-Rodríguez et al., 2019). In contrast, a previous study conducted in north Israel identified a much lower percentage of biofilm producers (53.6%, 66/123) among hospital-isolated strains (Rahmoun et al., 2021). It is possible that there are differences in biofilm-forming capacities of hospital vs. community isolates. Such variability may result from the differences in the type and duration of environmental stresses that bacteria encounter in hospital versus community, as one external stress is a major inducer to biofilm formation. Future studies should investigate this discrepancy.

The same former study in Israel among hospital-isolated strains classified ST2, ST4 and ST104 isolates as strong biofilm producers, which contrast the current findings in which all ST104 isolates (100%, 9/9) were weak biofilm producers (Rahmoun et al., 2021). Similarly, the majority of ST42 isolates were weak biofilm producers (92.3%, 12/13), whereas only a small proportion of ST2 isolates (5.9%, 1/17) demonstrated strong biofilm production capacities. This disparity may be attributed to the focus of the present work, which specifically examined CA-CDI, as opposed to the previous study, which investigated HA-CDI isolates. Taken together, STs may demonstrate diverse behaviors depending on their origin, whether acquired from the community or from a hospital setting. As mentioned above, this diversity may result from differences in the environment. To gain a more comprehensive understanding of this association, further investigations are warranted.

As for toxin production, 88.9% of the ST11 isolates included in the current analysis produced toxin B. TcdB is known to induce higher toxicity in human colonic epithelium compared to TcdA (Riegler et al., 1995; Aktories et al., 2017). ST11, usually the prevailing strain among animals, such as cattle, is more frequently detected in human CDI. This shift has been associated with severe disease manifestations and higher mortality rates, particularly within community settings (Goorhuis et al., 2008). Additionally, the identification of ST11 in piglets in China suggests a potential for zoonotic CDI risks in the community (Zhang et al., 2019).

In the current work, 35.3% of the ST2 isolates producing toxin A, 41.2% producing toxin B, and 23.5% producing both toxins. In contrast to the above, in the aforementioned study conducted in Israel on isolates obtained from hospitalized patients with CDI during 2017–2018, 7 none of the ST2 isolates produced toxin A, while 80% of the isolates produced both toxins A and B, and 20% produced only toxin B (Rahmoun et al., 2021).

In the previous study, none of the ST104 isolates produced toxin A. In comparison, in the current study, 10% of the ST104 isolates produced toxin A (Rahmoun et al., 2021). Furthermore, 66.7% of the ST104 isolates in the previous study produced both toxins, whereas in the current study, 50% of the isolates exhibited dual toxin production. In the previous study, 33.3% of the ST104 isolates produced toxin B, 40% of the isolates expressed toxin B. These findings further support the possibility of distinct characteristics depending the acquisition settings.

### 4.2 Susceptibility of community *Clostridioides difficile* isolates to antibiotics

In the current study, isolates demonstrated high susceptibility to both vancomycin and metronidazole (96.8% and 97.6%, respectively), which are the standard medications administered to treat CDI. Furthermore, no significant changes in susceptibility to these antibiotics were noted over the years. Studies conducted in different regions of the world reported 100% susceptibility to metronidazole and vancomycin (Jamal and Rotimi, 2016; Kociolek et al., 2016; Kullin et al., 2016; Thornton et al., 2018).

Due to the lack of cutoff values for fidaxomicin, the overall rate of resistance remains unknown. In the current study, the highest MIC observed was 32 μg/mL (5/126, 4%), which is still lower than the maximum MIC reported in the literature, i.e., 64 μg/mL (Schwanbeck et al., 2019).

Although high antibiotics susceptibility was noted, the limited number of isolates for specific did not allow for assessment of a possible association between ST and MIC. Nonetheless, it was noted that higher MIC values were measured for specific STs such as ST104 and ST11. Thus, further studies with larger numbers of isolates per each ST should pursue this issue.

In conclusion, the current study demonstrated a high diversity of STs in community CDIs in Israel, with no dominant strain. These findings align with those of other Western countries. The distribution of STs in various geographic areas is different and further studies should investigate the reasons for this variability. Another interesting finding is that STs may exhibit distinct characteristics depends on their origin- in the community or in healthcare facilities. Further investigation is needed to explore the factors that contribute to these differences. Finally, on the one hand, the CA-CDI strains that were presented in the current study were mostly biofilm and toxin producers, and on the other hand, they did not induce a high mortality rate as hospital strains. This evidence strengthens the idea that clinical outcomes are affected from both bacterial virulence and patients’ characteristics such as their immunity.

## Data availability statement

The data presented in the study were deposited in NCBI repository, accession number PRJNA1044122.

## Ethics statement

The studies involving humans were approved by POR-0085-15, WOMC-0115-20, SOR-0307-20. The studies were conducted in accordance with the local legislation and institutional requirements. The participants provided their written informed consent to participate in this study.

## Author contributions

OSc: Methodology, Validation, Writing – review & editing, Conceptualization, Data curation, Formal analysis, Investigation. HR: Data curation, Formal analysis, Investigation, Methodology, Validation, Writing – original draft, Writing – review & editing, Visualization. MA: Data curation, Formal analysis, Investigation, Project administration, Supervision, Validation, Visualization, Writing – original draft, Writing – review & editing. AS: Formal analysis, Investigation, Methodology, Validation, Writing – review & editing. NR: Formal analysis, Investigation, Methodology, Validation, Writing – review & editing. YM: Methodology, Validation, Writing – review & editing. LN: Methodology, Validation, Writing – review & editing. OSa: Methodology, Validation, Writing – review & editing. SK-D: Formal analysis, Methodology, Writing – review & editing. PK: Formal analysis, Methodology, Writing – review & editing. AP: Conceptualization, Formal analysis, Investigation, Project administration, Supervision, Validation, Visualization, Writing – original draft, Writing – review & editing.

## Footnotes

^1^ http://pubmlst.org/cdifficile/
